# Genomic Imprinting at the Porcine *PLAGL1* Locus and the Orthologous Locus in the Human

**DOI:** 10.3390/genes12040541

**Published:** 2021-04-08

**Authors:** Jinsoo Ahn, In-Sul Hwang, Mi-Ryung Park, Seongsoo Hwang, Kichoon Lee

**Affiliations:** 1 Functional Genomics Laboratory, Department of Animal Sciences, The Ohio State University, Columbus, OH 43210, USA; ahn.134@osu.edu; 2 Animal Biotechnology Division, National Institute of Animal Science, Rural Development Administration, Wanju, Jeonbuk 55365, Korea; insuri2642@korea.kr (I.-S.H.); mrpark45@korea.kr (M.-R.P.); hwangss@korea.kr (S.H.)

**Keywords:** genomic imprinting, monoallelic expression, pig, DNA methylation, porcine, *PLAGL1*, transcriptome, topological boundary regions, CTCF, alternative splicing

## Abstract

Implementation of genomic imprinting in mammals often results in *cis*-acting silencing of a gene cluster and monoallelic expression, which are important for mammalian growth and function. Compared with widely documented imprinting status in humans and mice, current understanding of genomic imprinting in pigs is relatively limited. The objectives of this study were to identify DNA methylation status and allelic expression of alternative spliced isoforms at the porcine *PLAGL1* locus and assess the conservation of the locus compared to the orthologous human locus. DNA methylome and transcriptome were constructed using porcine parthenogenetic or biparental control embryos. Using methylome, differentially methylated regions between those embryos were identified. Alternative splicing was identified by differential splicing analysis, and monoallelic expression was examined using single nucleotide polymorphism sites. Moreover, topological boundary regions were identified by analyzing CTCF binding sites and compared with the boundary of human orthologous locus. As a result, it was revealed that the monoallelic expression of the *PLAGL1* gene in porcine embryos via genomic imprinting was maintained in the adult stage. The porcine *PLAGL1* locus was largely conserved in regard to maternal hypermethylation, tissue distribution of mRNA expression, monoallelic expression, and biallelic CTCF-binding, with exceptions on transcript isoforms produced by alternative splicing instead of alternative promoter usage. These findings laid the groundwork for comparative studies on the imprinted *PLAGL1* gene and related regulatory mechanisms across species.

## 1. Introduction

Implementation of genomic imprinting in mammals during gametogenesis often results in *cis*-acting silencing of nearby genes in a cluster [1]. In addition, when the use of alternative promoters coincides with adjacent genomic imprints, multiple transcripts are produced that are imprinted in a transcript-specific manner [2]. For example, the *GNAS* locus has been shown to harbor both maternal and paternal differentially methylated regions (DMRs) which serve as the imprinting control region (ICR), and concurrent alternative promoter usage led to isoform-dependent parental-origin-specific expression [3,4]. Our group has identified a combinatorial imprinting pattern in the *GNAS* locus in pigs which consists of either maternal, paternal, or biallelic expression of transcripts in relation to corresponding DMRs [5]. To make it more complex, tissue-specific or non-tissue-specific as well as time-dependent or time-independent imprinting patterns coexist [6]. Consequently, the resulting monoallelic or allele-specific expression (or, allelic imbalance) plays an important role in mammalian growth, development, tissue function, and phenotypic plasticity [7,8]. Compared with widely documented imprinting status in humans and mice, current understanding of genomic imprinting in pigs is relatively limited; therefore, our attempts were made to delineate imprinting in porcine loci using parthenogenetic embryos, and whole-genome bisulfite sequencing (WGBS), and RNA-sequencing (RNA-seq) [5,9].

The PLAG1 like zinc finger 1 (*PLAGL1*) gene is also known as pleiomorphic adenoma gene-like 1 [10], a zinc finger protein which regulates apoptosis and cell cycle arrest 1 (*ZAC1*) [11,12], and lost-on-transformation 1 (*LOT1*) [13,14,15]. This gene encodes a transcription factor with seven C_2_H_2_-type zinc fingers that induces apoptosis through DNA binding and transcriptional coactivation of p53, and promotes cell cycle arrest [11,16,17]. The loss or reduction of *LOT1/ZAC1/PLAGL1* expression in several types of cancers and its proapoptotic and cell cycle arresting properties have suggested its potential role as a tumor suppressor [15,18]. Suppression of *PLAGL1* expression during tumor metastasis has been proposed to be related to transcriptional repression through the recruitment of methyltransferase and aberrant DNA methylation in the promoter region [17]. With regard to nondiseased embryonic and adult human tissues, the expression of *PLAGL1* is ubiquitous, with a higher degree in placenta and a lesser degree in the whole brain, liver, and skeletal muscle, according to previous studies [12,15], the Human Protein Atlas (HPA) [19], and Genotype-Tissue Expression (GTEx) project [20]. This normal expression is epigenetically regulated by maternal methylation imprints which force a paternal monoallelic expression of *PLAGL1* in humans and mice [21,22,23], with an exception of biallelic expression in the mouse liver [22]. On the other hand, paternal duplication of the *PLAGL1* locus is responsible for transient neonatal diabetes mellitus (TNDM) which has characteristics such as intrauterine growth retardation and hyperglycemia due to lack of normal insulin secretion [24]. 

In pigs, studies on the *PLAGL1* locus and its imprinting status are limited. DNA methylation status at the porcine *PLAGL1* locus including putative promoter regions, which might regulate monoallelic expression or biased allelic expression, has remained unexplored. It was previously shown that expression of *PLAGL1* was almost equal in multiple tissues based on semiquantitative PCR, but not including the brain [25], and a paternal expression of *PLAGL1* was reported based on PCR-RFLP targeting the overlapping last exon, but not by distinguishing transcript isoforms [26]. Thus, in-depth quantification in major tissues and analyses on isoform-dependent monoallelic expression have not been accomplished. Of note, large local chromatin interaction domains, termed topological domains, are highly conserved across mammalian species, and the boundaries of topological domains are strongly enriched with CTCF, the insulator binding protein [27]. As such, CTCF is a key regulator for organizing chromosomal interactions, territories, and structure. A previous study reported topological boundary regions in the human and mouse *PLAGL1* locus in a comparative manner [23]; however, to our knowledge the boundary and its conservation in pigs have not been investigated. 

In this study, DNA methylome and transcriptome obtained from our porcine parthenogenetic and biparental control embryos were used to identify maternal DMRs and corresponding paternal monoallelic expression of alternatively spliced isoforms within the *PLAGL*1 locus. In adult pigs, a difference in expression levels of *PLAGL*1 in major tissues and its alternative splicing were analyzed using public RNA-seq data, and also monoallelic expression of each transcript isoforms were examined by combining public whole-genome sequencing (WGS) data and corresponding transcriptome. Furthermore, topological boundary regions in porcine *PLAGL1* locus were identified and compared with the boundary in the orthologous locus of the human. Consequently, a detailed and comprehensive overview of *PLAGL1* locus in pigs was generated that can advance our understanding on this locus in a cross-species comparative manner.

## 2. Materials and Methods

### 2.1. Animal Ethics Statement

Animal procedures used in the current study were approved by the Institutional Animal Care and Use Committee (IACUC) of the National Institute of Animal Science, Rural Development Administration (RDA) of Korea (approval number NIAS2015-670).

### 2.2. Sample Acquisition

Oocytes from Landrace × Yorkshire × Duroc (LYD) pigs were collected and matured in vitro. As described in our previous reports [5,9], parthenogenetic embryos were generated by electrical stimulation of oocytes. Those parthenogenetic embryos were developed for 21 days after placing into oviducts of surrogate gilts. As experimental controls, fertilized embryos were collected at day 21 from LYD gilts after natural mating as previously described in our publications [5,9].

### 2.3. Whole-Genome Bisulfite Sequencing (WGBS)

Genomic DNA was isolated from control (CN, *n* = 3) and parthenogenetic (PA, *n* = 3) whole embryos and subjected to WGBS as reported previously [5,9]. In brief, based on the manufacturer’s instructions, bisulfite conversion of genomic DNA was performed using Accel-NGS Methyl-Seq DNA Library Kit (Swift Biosciences, Inc., Ann Arbor, MI, USA). Adapter primers and Diastar™ EF-Taq DNA polymerase (Solgent, Daejeon, Korea) were used to perform PCR under thermal conditions as follows: an initial denaturation (3 m at 95 °C), 35 cycles of 30 s at 95 °C, 30 s at 60 °C, and 30 s at 72 °C, and a final extension for 5 m at 72°C. PCR products (151 nt paired-end) were sequenced, after bead-based clean-up, using an HiSeqX sequencer operated by Macrogen Inc. (Seoul, Korea). Quality check of the raw reads was done by FastQC (v0.11.7) and following adapter trimming and filtering out reads shorter than 20 bp were conducted using Trim Galore (v0.4.5). The numbers of remaining cleaned reads were 846.5 (CN1), 862.1 (CN2), 866.5 (CN3), 839.7 (PA1), 856.9 (PA2), and 849.2 (PA3) million. BSMAP aligner (v2.87) [28] was used to map the cleaned reads to the pig reference genome (Sscrofa11.1) and extract the methylation ratio of every CpGs.

### 2.4. RNA Sequencing (RNA-Seq)

RNA-seq was performed to produce transcriptome as described [5,9]. Total RNA was isolated from the whole collected CN and PA embryos (*n* = 3, each) using TRIzol reagent (Sigma-Aldrich, St. Louis, MO, USA) following the manufacturer’s instruction. Briefly, RNA was treated with DNase I to avoid genomic DNA contamination and electrophoresed in 1.2% agarose gels to evaluate the integrity. The RNA quality was further confirmed by the 28S/18S rRNA ratio more than 2.0 and the RNA integrity number (RIN) more than 7.0 using an Agilent 2100 BioAnalyzer. The concentration of RNA was assessed using the ratios of A260/A280 and A260/A230 (1.8–2.0). To construct cDNA libraries with the TruSeq RNA Sample Prep Kit v.2 (Illumina, San Diego, CA, USA), 1 ug of total RNA was used. The cDNA libraries were quantified by quantitative Real-Time PCR (qPCR) and qualified by the Agilent 2100 Bioanalyzer, and then, the library products (101 nt paired-end) were sequenced by the Illumina HiSeq2500 platform. After checking the quality of the raw data by FastQC, reads were adapter-trimmed and filtered by Trim Galore, leaving ~76.8 (CN1), 73.0 (CN2), 77.2 (CN3), 80.0 (PA1), 79.3 (PA2), and 80.3 (PA3) million cleaned reads. Those cleaned reads were aligned to the pig reference genome (Sscrofa11.1) using STAR aligner (v.2.7.5) [29] with default parameter settings. The aligned reads in BAM files were further processed by deepTools (v3.5.0) [30] to normalize read coverages.

### 2.5. Mining and Processing RNA-Seq, Whole-Genome Sequencing (WGS), Variant Call Format (VCF), and ChIP-Seq Data

Raw data generated by RNA-seq (150-bp reads, paired-end) with samples from seven tissues (adipose tissue, brain, liver, lung, skeletal muscle, heart, and ovary) of adult female pigs (n = 3, 180-day-old Large White gilts) and WGS (150–bp reads, paired-end) with skeletal muscle samples from each gilt were retrieved from the FAANG project under accession number PRJNA493166 (https://data.faang.org/dataset/PRJNA493166; accessed on 21 January 2021) [31]. These raw RNA-seq data were processed using the same software as above (FastQC, Trim Galore, STAR aligner, and deepTools), to analyze tissue distribution and monoallelic expression of the PLAGL1 transcripts. The raw WGS data were checked for quality and trimmed using FastQC and Trim Galore, and then, bwa-mem aligner (v0.7.17-r1198) was used to align the cleaned reads to the pig reference genome (Sscrofa11.1). Those aligned reads were used to identify single nucleotide polymorphisms (SNPs) in the exonic regions of the PLAGL1 gene.

Published SNP data in VCF format were retrieved from the European bioinformatics institute (EBI) FTP site (http://ftp.ebi.ac.uk/pub/databases/eva/rs_releases/release_2/by_species/sus_scrofa/Sscrofa11.1/GCA_000003025.6_current_ids.vcf.gz; accessed on 14 November 2020). CTCF ChIP- seq data from human normal lymphoblast cells under accession number GSE155324 (https://www.ncbi.nlm.nih.gov/geo/query/acc.cgi?acc=GSE155324; accessed on 19 February 2021) (bw files, human reference genome (hg19)) and from pig embryonic fibroblasts (PEFs) isolated from 35-day-old fetuses of Large White pigs under GSE153451 (https://www.ncbi.nlm.nih.gov/geo/query/acc.cgi?acc=GSE153451 ; accessed on 10 January 2021) (FASTQ files) were obtained from the NCBI GEO repository. The raw FASTQ reads were cleaned, aligned as above using bwa-mem, and normalized using deepTools.

### 2.6. DMR Calling

The program metilene (v0.2-8) [32] was used to call a DMR with thresholds as follows: at least 10 differentially methylated CpGs with an averaged methylation ratio difference (∆ ave) more than 0.2, distance between those CpGs less than 300 bp, and false discovery rate (FDR) < 0.05.

### 2.7. Differential Splicing Analysis

Differential splicing (DS) was analyzed on RNA-seq data using two types of DS analysis tools adapting isoform-based or event-based approaches [33]. The isoform-based analysis was performed via transcriptome quantification by Kallisto [34] using raw FASTQ reads and ensuing differential analysis by Sleuth [35]. Adjusted *p*-value < 0.05 was set as a statistical significance. To confirm differential alternative splicing, one of the event-based methods, rMATS [36] was used. 

### 2.8. Data Visualization

WGBS, RNA-seq, WGS, and ChIP-seq data were visualized using the R/Bioconductor package Gviz (v1.32.0) [37] and/or Integrative Genomics Viewer (IGV) (v2.8.13) [38]. The CRAN package ggplot2 was used to display DS analysis results. 

## 3. Results

### 3.1. The PLAGL1 Locus in Pig Embryos Harboring Maternally Imprinted and Paternally Expressed Alternatively Spliced Transcripts

**Figure 1:**
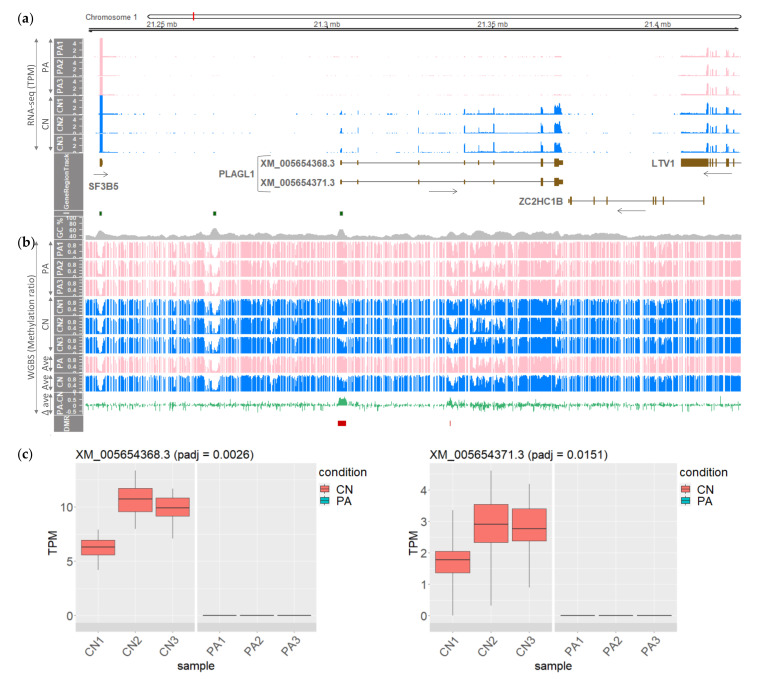
Paternal monoallelic expression of spliced isoforms of the porcine *PLAGL1* gene in CN embryos, and maternal DNA hypermethylation in PA embryos. (**a**) Normalized RNA-seq read coverages in TPM are presented throughout the loci containing two *PLAGL1* transcripts: XM_005654368.3 with eight exons and XM_005654371.3 with exon-5 skipping. Left panel: PA, parthenogenetic embryos; CN, control embryos; GeneRegionTrack, track containing protein-coding genes (tall box, translated region; short box, untranslated region; horizontal arrow, direction of transcription); I, CpG island; GC%, GC content in a percentage. (**b**) Methylation ratios on CpGs obtained by WGBS are displayed at single-base resolution. Left panel: Ave, averaged methylation ratio; ∆ ave, delta average subtracting CN ave from PA ave; DMR, differentially methylated region (FDR < 0.05). (**c**) Transcript-level quantification followed by differential expression analysis resulted in identification of a major transcript (left) and one alternatively spliced isoform with exon-5 skipping (right) (padj, adjusted *p*-value, <0.05).

To delineate the link between monoallelic paternal expression of the porcine *PLAGL1* gene and DNA methylation status, first, two different expression types of *PLAGL1* in biparental control embryos and parthenogenetic (unimaternal) embryos were compared. It was shown that *PLAGL1* expression was exclusive in control embryos having a paternal allele; whereas, paternal-allele-absent parthenogenetic embryos lacked *PLAGL1* expression, indicating a paternal monoallelic expression of *PLAGL1* in an embryonic stage (Figure 1a). Using a difference in the methylation ratios, two DMRs that were maternally hypermethylated in PA embryos were detected: one encompassing the promoter region, exon 1, and the CpG island, and the other in between exons 3 and 4 (Figure 1b). In both CN and PA embryos, hypomethylation was shown inchr1:21335000-21355000, which surrounds exons 4–6 of XM_005654368.3, and also in the last exon (Figure 1b). Within the locus between the porcine *UTRN* and *PEX3* genes in 1-Mb region (chr1: 20800000-21800000), the paternal expression was detected only in the *PLAGL1* gene and the DMRs were located only in the *PLAGL1* locus (Figure S1).

Moreover, out of all 30 annotated *PLAGL1* transcripts in the NCBI Gene (https://www.ncbi.nlm.nih.gov/gene/733596; accessed on 5 January 2021) and Ensembl (https://useast.ensembl.org/Sus_scrofa/Gene/Summary?g=ENSSSCG00000004127; accessed on 5 January 2021) databases, bona fide alternatively spliced transcripts were identified as follows: transcript-level quantification by Kallisto [34] and ensuing differential expression analysis by Sleuth [35] revealed two statistically significant isoforms (XM_005654368.3 and XM_005654371.3) that are differentially expressed in CN embryos compared to PA embryos (Figure 1c). Normalized read coverages in transcripts per million (TPM) of XM_005654368.3 were approximately 3-fold higher than those of XM_005654371.3 in all three CN embryos. In comparison with the major XM_005654368.3 form, exon-5 skipping was carried out in the minor XM_005654371.3 form whose skipped exon (SE) was verified (FDR = 0.0546) by rMATS [36], a method for detecting differential alternative splicing events. In summary, both the two *PLAGL1* transcripts were paternally expressed in CN embryos and maternally repressed in PA embryos, due probably to a direct silencing of maternal alleles through maternal hypermethylation in the promoter region.

### 3.2. Differentially Methylated CpGs within the PLAGL1 Locus in the Porcine Genome

**Figure 2:**
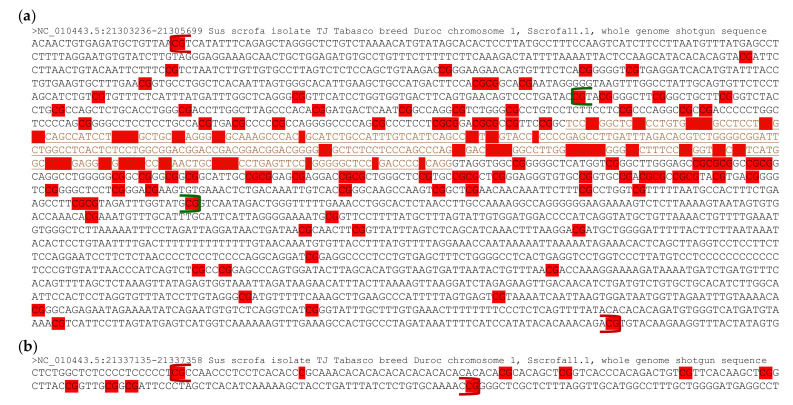
Distribution of differentially methylated CpGs in the two DMRs at the *PLAGL1* locus of pig embryos. (**a**) The promoter region (upstream), exon 1 (brown, underlined), and intronic region (downstream) of the two spliced isoforms in Figure 1 are displayed at chr1 or NC_010443.5: 21303236-21305699. Red highlighted letters denote differentially methylated CpG dinucleotides or Gs (i.e., maternally hypermethylated cytosines in PA embryos). Gs indicate methylated Cs on the minus strand (coding strand). The beginning and end of DMR (chr1:21303256–21305675) are marked with red brackets, within which green brackets indicate the CpG island (chr1:21303765–21304604). (**b**) The intronic region between exons 3 and 4 (chr1 or NC_010443.5:21337135-21337358) of the two spliced isoforms is presented. The DMR, denoted with red brackets, holds 10 differentially methylated CpGs or C. Thresholds for DMR calling are stated in Section 2.

As DNA hypermethylation is involved in inhibition of tumor suppressor genes [39], a profiling of maternal DNA hypermethylation within the two DMRs was conducted in detail in the *PLAGL1* locus. Partial genomic DNA sequences from chromosome 1 of the pig reference genome (Sscrofa11.1) were retrieved from the NCBI Nucleotide database (https://www.ncbi.nlm.nih.gov/nuccore; accessed on 7 January 2021). The CpG island, overlapping the proximal *PLAGL1* promoter (aka, promoter P1) and the corresponding exon 1 in the human and mouse [23], is conserved as the CpG island (840 bp) in pigs spans between the promoter, transcription start site (TSS), 1st exon, and upstream of the intronic region (Figure 2a). The 1st *PLAGL1* DMR in pigs encompassed between farther upstream of the promoter and downstream of the intronic region with sparse regions, similar to the *PLAGL1*/*HYMAI* DMR in the human and mouse [23] (Figure 2a, Table S1). Differentially methylated CpGs or Gs (Cs on the coding strand) between PA and CN embryos were denser in the CpG island than up- or downstream, suggesting the importance of this CpG island in epigenetic gene regulation through DNA methylation. The second *PLAGL1* DMR in pigs consisted of 10 differentially methylated CpGs or Cs which satisfied one of the criteria for DMR calling (Figure 2b). In summary, the CpG island is conserved across the human, mouse, and pig, and differentially methylated CpGs were concentrated on the CpG island which is surrounded by the 1st DMR in pig embryos.

### 3.3. Tissue Distribution and Monoallelic Expression of the PLAGL1 Gene in Adult Pigs

**Figure 3:**
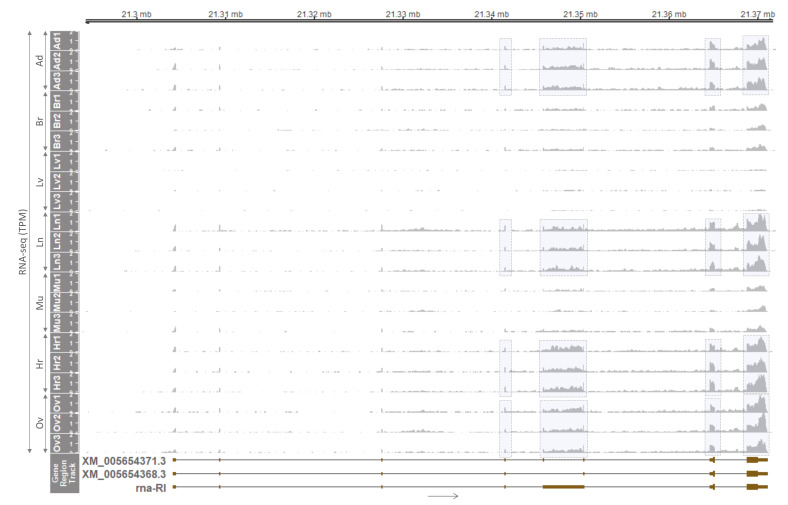
Expression profiling of spliced isoforms originated from the porcine *PLAGL1* gene in an adult stage (180-day-old) based on RNA-seq data. In GeneRegionTrack, two transcripts (XM_005654368.3 and XM_005654371.3) and another transcript with intron retention at between exons 5 and 6 (rna-RI) are displayed. Grey boxes indicate a higher level of expression in corresponding exons in Ad, Ln, Hr, and Ov tissues compared to Br, Lv, and Mu tissues. Additionally, read coverages in these grey boxes were analyzed for monoallelic expression on SNP sites as below in Figure 4. Triplicates of each tissue were derived from three different female adult pigs. Raw RNA-seq data under PRJNA493166 were retrieved from the FAANG project and processed as in Section 2. Ad, adipose tissue; Br, brain; Lv, liver; Ln, lung; Mu, skeletal muscle; Hr, heart; Ov, ovary.

In order to investigate whether monoallelic expression pattern maintains in a later developmental stage, the expression of the *PLAGL1* gene in adult pigs was examined. First, tissue distribution of the expression was explored using RNA-seq data from female pigs under accession number PRJNA493166 [31]. After quality control and read alignment, RNA-seq read coverages were displayed throughout every exon of the major transcript (XM_005654368.3) and its alternatively spliced isoform (XM_005654371.3). Read coverages were relatively higher in adipose tissue, lung, heart, and ovary, than in the brain and skeletal muscle, while the expression was almost nondetectable in the liver (Figure 3). As mentioned above, a lesser degree of expression in the human whole brain, liver, and skeletal muscle was reported in literature, HPA, and GTEx project, and in the current study, the low expression in those three tissues was conserved in pigs. Noticeably, in between exons 5 and 6 of the major transcript, intron retention was also observed, and a corresponding transcript was denoted as rna-RI (RI stands for a retained intron) (Figure 3). Four exonic regions in those four tissues (adipose tissue, lung, heart, and ovary) having higher read coverages were highlighted with grey boxes and selected to further examine allelic expression.

**Figure 4:**
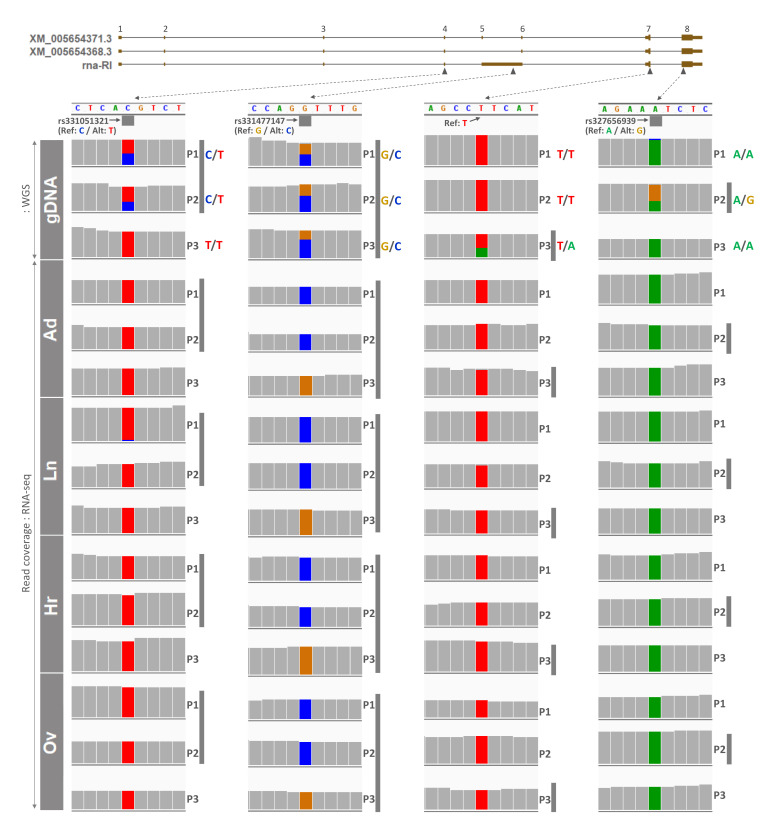
Informative SNPs and monoallelic expression of alleles from the *PLAGL1* gene in adult pigs. Below exons of transcripts, black filled triangles point out each SNP, from which dotted arrows are connected to partial nucleotide sequences from the pig reference genome (Sscrofa11.1). Grey filled squares under the nucleotide sequence indicate published SNPs with rs ID (reference SNP ID) and genotypes (Ref, reference allele; Alt, alternative allele) that were obtained from the EBI FTP site. A SNP in exon 7 was not previously reported, and only a reference allele from the genome is denoted. Read coverages from matched WGS and RNA-seq raw data from PRJNA493166 were processed and are displayed on each nucleotide. Grey perpendicular bars indicate either heterozygous SNPs (informative) in genomic DNA from skeletal muscle (gDNA track) or monoallelic expression in cDNA from each tissue (Ad, adipose tissue; Ln, lung; Hr, heart; Ov, ovary) from pigs. P1, adult pig 1; P2, adult pig 2; P3, adult pig 3. Each allele in letters and read coverages is coded with the same color. The gDNA tracks without grey perpendicular bars indicate either a homozygous SNP (noninformative), rs331051321, in P3 or expression of a reference allele, followed by corresponding allele expression in each tissue shown in the tissue tracks.

Next, raw WGS data under PRJNA493166 [31], generated using genomic DNA isolated from skeletal muscle of adult female pigs 1 through 3 (P1-–3), were processed to screen heterozygous SNPs (informative) in those four exonic regions. In exon 4, retained intron (RI), and exon 8 (last exon), three published SNPs were found to be heterozygous or informative (Figure 4). In particular, rs331051321 was heterozygous (C/T) in P1 and P2, rs331477147 was heterozygous (G/C) in all three pigs, and rs327656939 was heterozygous (A/G) in P2. In exon 7, nonreported informative SNP (T/A) was found in P3 (Figure 4). Then, allelic expression at those SNP sites in cDNA from tissues of the same pigs were examined using the aforementioned RNA-seq data (Figure 3). Read coverages were plotted for each nucleotide, and allelic expressions on SNP sites were highlighted on both letters and read coverages with four different colors for A, T, G, and C. At the site of rs331051321 in overlapping exon 4, all four tissues from P1 and P2 were subjected to expression of only the alternative allele (T), except lung tissue from P1 having 5% reference allele and 95% alternative allele expression (Figure 4 and Table 1). Additionally, in other overlapping exons 7 and 8, on both nonreported SNP and rs327656939, respectively, reference alleles (T in P3, and A in P2) were expressed 97–100% (Figure 4 and Table 1). Taken together, in those three overlapping exons, expression of either the alternative or reference allele indicated monoallelic expression at those SNP sites with small and nonsignificant variations. These triplicated results on overlapping exons also indicated that all possible *PLAGL1* transcripts derived from the locus, including the displayed three isoforms, were monoallelically expressed, or expression of other transcripts besides the three isoforms was very low or negligible. Furthermore, nonoverlapping retained intron (RI) was also subjected to monoallelic expression of either the alternative allele (P1 and P2) or reference allele (P3) at 100% frequency (Figure 4 and Table 1). Overall, expression of *PLAGL1* was selectively high in certain tissues, and allelic expression in nonoverlapping RI was perfectly monoallelic, while in overlapping exons monoallelic expression was slightly degraded, but only at a nonsignificant level, suggesting maintenance of monoallelic expression of *PLAGL1* during development throughout the adult stage.

### 3.4. Conservation of CTCF Boundaries and Sequence Elements in Humans and Pigs

**Figure 5:**
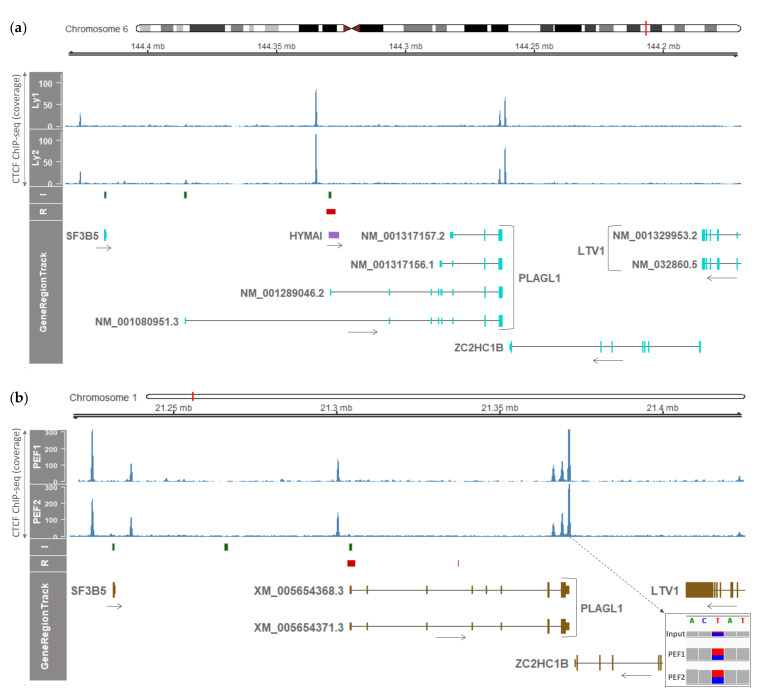
Topological boundary regions in the human and porcine *PLAGL1* locus. (**a**) The human *PLAGL1* locus in an orientation of downstream (left) to upstream (right). CTCF ChIP-seq data (accession number GSE155324) from two human lymphoblasts (Ly1 and 2) showed a previously reported 70-kb boundary surrounding the *HYMAI* transcript and three *PLAGL1* transcripts (NM_001289046.2, NM_001317156.1, and NM_001317157.2). A purple box represents a noncoding transcript, while protein-coding human transcripts are denoted with boxes in light blue. (**b**) The pig *PLAGL1* locus in an orientation of upstream (left) to downstream (right). CTCF ChIP-seq data (accession number GSE153451) from two pig embryonic fibroblasts (PEF1 and 2) displayed CTCF enrichment sites. Potential topological boundary regions encompassing the two *PLAGL1* transcripts in the embryonic stage were approximately 52-kb apart. A box in the right bottom showed a biallelic precipitation via CTCF binding immediate downstream of the last exon in both PEF1 and PEF2, as an allele in the input was heterozygous (T/C, T:25%, C:75%). I, CpG island; R, DMR.

Previously, in both humans and mice, it was reported that the imprinted transcripts from the *PLAGL1* transcriptional unit are restricted within the topological boundary regions enriched with CTCF [23]. Since this restriction suggested a conserved regulatory function of topological boundary regions, CTCF ChIP-seq data from human normal lymphoblast cells and pig embryonic fibroblasts (PEFs) were compared to identify potential topological boundary regions in pigs and its conservation with the human and mouse. First, in the human normal lymphoblast cells, the locus containing four previously detected *PLAGL1* transcript isoforms [23] and the noncoding transcript, *HYMAI*, was examined. For better comparison with the porcine *PLAGL1* locus, the human genomic sequence was reversed using reverseStrand function in R/Bioconductor package Gviz. As expected based on a previous report in the human [23], 70-kb apart topological boundary regions in downstream of the *PLAGL1*/*HYMAI*-DMR and within/upstream of the last exon were detected by enrichment of CTCF in lymphoblasts (Figure 5a). As also reported, there were two independent CTCF peaks within/upstream of the last exon (Figure 5a). In addition, this previous report showed that biallelic precipitation through CTCF binding occurred in the human *PLAGL1* 3′ UTR. 

Next, in PEFs, based on the two PLAGL1 transcripts (XM_005654368.3 and XM_005654371.3) detected in the pig embryonic stage in this report, CTCF was found to be enriched upstream of the 1st exon and within/downstream of the last exon, indicating that topological boundary regions are conserved in pig embryos (Figure 5b). The gap between the potential topological boundary regions was approximately 52 kb, which was narrower than the human one (~70 kb) and wider than the mouse one (~40 kb) [23]. The CpG island and the first DMR were located at the position similar to that of the human ones, within the boundary (Figure 5b). Additionally, similar to the human lymphoblast, biallelic precipitation via CTCF binding was detected in both PEFs, as genomic DNA contained in the input showed a heterozygous allele at immediate downstream of the last exon and precipitate alleles from both PEF1 and 2 were also heterozygous (PEF1, T:88 (58%)/C:65 (42%); PEF2, T:222 (53%)/C:193 (47%)) (Figure 5b). As CpG dinucleotides surrounding those CTCF binding sites were hypomethylated in human leucocytes and placenta [23], this biallelic precipitation in PEFs suggested hypomethylation in both alleles in the last exon (Figure 1b) might allow biallelic CTCF binding. Regarding the CTCF binding region immediate upstream of the 1st exon, there was no heterozygous allele. To sum up, topological boundary regions, sequence elements such as the CpG island, and biallelic binding of CTCF were conserved in the pig embryonic fibroblasts, indicating that inherent properties of these components are important across mammalian species.

## 4. Discussion

The findings in this study showed that the porcine *PLAGL1* locus contains the maternally hypermethylated DMR that drives a paternal monoallelic expression, and this monoallelic expression through genomic imprinting at this locus in the embryonic stage was maintained in the adult pigs. It was also shown that the epigenetic regulatory elements and phenomena that regulate the *PLAGL1* locus in the human and mouse are largely conserved in pigs with exceptions. One of these conservations was maternal hypermethylation in the region containing the CpG island and the first exon in the human and mouse [21,22,23] which was previously unidentified in pigs. By analyzing differential methylation between PA embryos (having two maternal alleles) and CN embryos (with one paternal and one maternal allele), hypermethylation in PA embryos was found which might be derived from methylation on the extra maternal allele, that is ‘maternal hypermethylation’. The corresponding DMR could be linked to inhibition of *PLAGL1* expression in PA embryos through transcriptional repression (i.e., a direct silencing of targeted loci), without affecting neighboring genes between *UTRN* and *PEX3* in pigs, similar to the orthologous locus in the human [23] (Figure 1 and Figure S1). This type of nonclustered imprinting was also reported in the human *PLAGL1* locus and termed a ‘microimprinting’ phenomenon that is presumably attributed to a nonsharing of *cis*-acting regulatory elements among neighboring genes [23]. On the other hand, the *Plagl1* gene has been shown to not only be epigenetically regulated, but also regulate expression of other imprinted genes such as paternally expressed *Igf2* and maternally expressed *H19* via producing a transcription factor, PLAGL1, that binds to the enhancer shared by both *Igf2* and *H19* genes (i.e., involved in a long-range communication between the enhancer and the promoter). Through this *trans*-acting property, *Plagl1* constitutes so-called ‘imprinted gene networks’, which might be established during mammalian evolution and take part in the regulation of embryonic growth [40,41]. In short, *PLAGL1* was maternally repressed in pigs via a direct silencing by conserved maternal hypermethylation in a narrow range in *cis*, and the paternally expressed imprinted transcription factor PLAGL1 can potentially affect a long-range communication between enhancers and promoters in other imprinted genes in *trans*.

According to the parental conflict theory in which paternally expressed genes favor increased growth at the expense of resources; whereas, maternally expressed genes accommodate growth reduction for resource reservation [42,43,44], involvement of paternally expressed imprinted *PLAGL1* in embryonic growth promotion can be justified and indeed *Zac1/Plagl1*-deficient mouse embryos showed growth restriction [40]. This growth promotion might be accomplished through the imprinted gene networks as mentioned above, although *PLAGL1* was first discovered with its proapoptotic and antiproliferative molecular properties [11,12] and regarded as a tumor suppressor gene [15,18]. Nevertheless, this hypothetical theorem for imprinted paternal expression of tumor suppressor genes towards growth may not be generally applicable to other tumor suppressor genes, given that *Igf2r* is a maternally expressed imprinted gene in mice and inhibits embryonic growth [45] and *DIRAS3* is a paternally expressed imprinted gene in humans (and in pigs, submitted for publication by our group) and also hinders growth [46,47,48], and both counteract the effects of growth promoting *Igf2* and *RAS*, respectively. 

Besides the conserved paternal expression of *PLAGL1*, among other conservations are tissue distribution of *PLAGL1* expression and non-tissue-specific monoallelic expression. Although a previous study reported expression variations in different brain regions [12], low expression in whole brain, liver, and skeletal muscle in pigs described in the same report was conserved in the current study. In regard to tissues expressing *PLAGL1* substantially, monoallelically expressed alleles were consistent across tissues in the same pigs. Therefore, this *PLAGL1* gene was unrelated to previously reported reversed allelic expression in the cases of the following genes: *SPTY2D1* in cattle (maternal allele expression in the brain caudal lobe and cerebellum, and paternal allele expression in kidney and thymus) [49] and *GRB10/Grb10* in humans and mice (maternal allele expression in placental trophoblast and paternal allele expression in the brain maintaining through adulthood due to alternative promoter usage) [50]. In this study, the placenta was unexplored where *PLAGL1* is expressed at a high level in the human [12,19], but according to a previous study [40], *Zac1/Plagl1*-deficient mice maintained normal placental function. In pigs, one SNP in the last exon was associated with shoulder fat thickness (SFT) and internal fat rate (IFR) [25], implicating roles of *PLAGL1* in adipose tissue metabolism. 

In addition, aforementioned conservations of topological boundary regions and sequence elements including the CpG island (Figure 5), biallelic hypomethylation at the boundary which is within/adjacent to the last exon (Figure 1b), and biallelic CTCF binding to the boundary region (Figure 5b) support the hypothesis that these topological domains evolved to mediate *cis*-regulatory roles in mammalian growth. One exceptional feature is that in the human and mouse locus, more transcripts that showed a comparable amount of mRNA expression, than in the pig locus, were reported including noncoding RNA (*HYMAI*) due to alternative promoter usage [15,23,51]. According to previously proposed models [23,52], biallelic CTCF-binding sites physically interact to form a chromatin loop and an additional second loop is created to bring transcription factors for activation of promoters located in the middle region of the locus. In pigs, since the two transcripts in the embryonic stage and the three transcripts in the adult stage shared the promoter region and TSS, the single chromatin loop may be sufficient for transcriptional activation instead of forming the additional second loop. This chromatin structure will need to be elucidated in future studies. 

## 5. Conclusions

In pigs, the monoallelic expression of the *PLAGL1* gene in the embryonic stage via genomic imprinting at the corresponding locus was maintained in the adult stage. Compared to the human orthologous locus, the porcine *PLAGL1* locus was conserved to a great extent, in terms of a maternally hypermethylated DMR, nonclustered microimprinting, tissue distribution of mRNA expression, consistent monoallelic expression, sequence elements, and the biallelic CTCF-binding boundary region. Exceptionally, alternative splicing events (exon skipping and intron retention) were identified in pigs, but not alternative promoter usage. Considering these splicing events, the single chromatin loop might be sufficient for transcriptional activation in the porcine *PLAGL1* locus, unlike the human orthologous locus. Future comparative studies across species focusing on chromatin conformation and imprinted gene networks will further advance our understanding on the *PLAGL1* locus and related regulatory mechanisms.

## Figures and Tables

**Table 1:** Heterozygous SNPs (informative) in exon 4, RI, exons 7 and 8, and read counts of each allele.

Genomic	Rs Release			Pig	gDNA (WGS)		Ad (RNA-Seq)		Ln (RNA-Seq)		Hr (RNA-Seq)		Ov (RNA-Seq)	
Coordinate	rs ID	Ref	Alt	ID	Ref	Alt	Ref	Alt	Ref	Alt	Ref	Alt	Ref	Alt
chr1:21341446	rs331051321	C	T	P1	10 (45%)	12 (55%)	0	26	2 (5%)	35 (95%)	0	25	0	33
				P2	6 (38%)	10 (63%)	0	24	0	26	0	33	0	24
chr1:21349510	rs331477147	G	C	P1	7 (47%)	8 (53%)	0	19	0	11	0	35	0	22
				P2	10 (42%)	14 (58%)	0	11	0	11	0	30	0	20
				P3	12 (32%)	25 (68%)	22 (100%)	0	33 (100%)	0	37	0	19	0
chr1:21365021	NR	T	A	P2	9 (60%)	6 (40%)	51 (98%)	1 (2%)	46 (100%)	0	60	0	40	0
chr1:21368747	rs327656939	A	G	P3	8 (38%)	13 (62%)	37 (97%)	1 (3%)	48 (98%)	1 (2%)	52	0	63 (97%)	2 (3%)

Rs Release, reference SNP release; NR, not reported. Details in Figure 4 legend.

## Data Availability

Data supporting the results were downloaded from public repositories associated with PRJNA493166, GSE155324, and GSE153451 and can be found within Supplementary Materials.

## Supplementary Materials

The following are available online at https://www.mdpi.com/article/10.3390/genes12040541/s1, Figure S1: Gene expression and DNA methylation within the locus between *UTRN* and *PEX3* in pigs. Details regarding the axis and samples are in Figure 1. Table S1: Differentially methylated region (DMR) in the locus between the porcine *UTRN* and *PEX3* genes called by the metilene software.
