# Supplementary figures and images for: Genomic Imprinting at the Porcine PLAGL1 Locus and the Orthologous Locus in the Human
Source: Genes (Basel). 2021 Apr 8;12(4):541. doi: 10.3390/genes12040541 (PMC8069715; doi:10.3390/genes12040541)

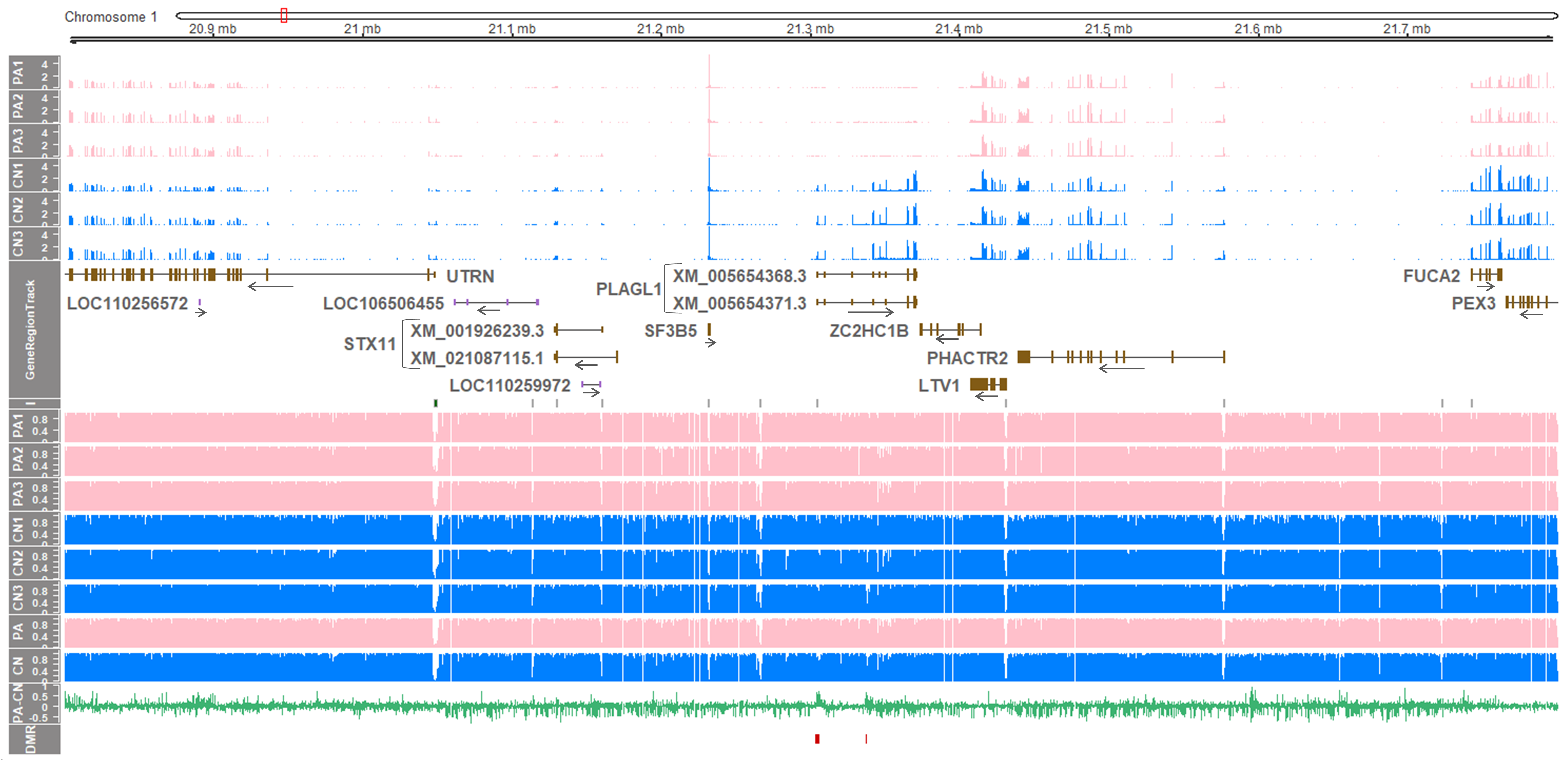

Supplement: Supplementary file 1 [file genes-12-00541-s001.zip › FigureS1.tiff]
